# Chemical Strain of Graphite-Based Anode during Lithiation and Delithiation at Various Temperatures

**DOI:** 10.34133/2021/9842391

**Published:** 2021-10-26

**Authors:** Zeyu Xu, Xiuling Shi, Xiaoqiang Zhuang, Zihan Wang, Sheng Sun, Kaikai Li, Tong-Yi Zhang

**Affiliations:** ^1^Materials Genome Institute, Shanghai University, 333 Nanchen Road, Shanghai 200444, China; ^2^School of Materials Science and Engineering, Harbin Institute of Technology, Shenzhen, China

## Abstract

Electrochemical lithiation/delithiation of electrodes induces chemical strain cycling that causes fatigue and other harmful influences on lithium-ion batteries. In this work, a homemade in situ measurement device was used to characterize simultaneously chemical strain and nominal state of charge, especially residual chemical strain and residual nominal state of charge, in graphite-based electrodes at various temperatures. The measurements indicate that raising the testing temperature from 20°C to 60°C decreases the chemical strain at the same nominal state of charge during cycling, while residual chemical strain and residual nominal state of charge increase with the increase of temperature. Furthermore, a novel electrochemical-mechanical model is developed to evaluate quantitatively the chemical strain caused by a solid electrolyte interface (SEI) and the partial molar volume of Li in the SEI at different temperatures. The present study will definitely stimulate future investigations on the electro-chemo-mechanics coupling behaviors in lithium-ion batteries.

## 1. Introduction

Although lithium-ion batteries (LIBs) are widely used in portable electronic devices, electric vehicles, and large-scale energy storage equipment nowadays, they are still facing significant challenges including unsatisfactory cycling performance and low energy densities especially in extended temperature ranges [1–4]. During charging/discharging cycling, lithium-ions reversibly or partially reversibly insert into and extract from the active materials of anode/cathode, resulting in a periodical volume change of the electrodes. According to theoretical calculations, the commercially used graphite anode experiences ~10% volume expansion after full lithiation (corresponding to a specific capacity of 372 mAh g^−1^) [5]. The volume expansion/contraction induced by lithiation/delithiation inevitably causes strain (stress) fatigue and might eventually initiate cracks in the active materials and/or composite electrodes due to the confinement of the current collector, which has been regarded as the major reason for degradation of LIB performance in terms of capacity and service life [6–9]. Thus, studying the electrochemical-mechanical coupling behavior of electrodes, including the investigation of electrochemical lithiation/delithiation-induced chemical strains in electrodes during cycling, is of great significance for the understanding of the electrochemical performance of LIBs and for the optimized design of electrodes and batteries [10–14].

Towards this aspect, researchers have developed several methods to study chemical strains/stresses in electrodes during electrochemical cycling, including mainly the digital image correlation (DIC) technique and the curvature-measurement method. Combining these experimental methods with theoretical models [12–14], the lithiation-induced stress/strain variation in the composite electrodes and even active materials can be quantitatively analyzed [11, 15, 16]. Generally, the chemical stress evolution with Li concentration is obtained by measuring in situ the curvature of a thin film electrode deposited on a substrate [11, 14]. The substrate prevents the Li-induced in-plane expansion/contraction of the film electrode during (de)lithiation, thereby resulting in the compressive (tensile) stress, which bends the film/substrate [17]. Li et al. measured the curvature change in silicon electrode film/copper substrate and in graphite electrode film/copper substrate, analyzed the chemical stress and the variation in elastic modulus of these electrodes, and found that the elastic modulus and stress of electrodes were dependent on the Li concentration [18, 19]. Kumar et al. and other researchers monitored the curvature changes in Si- [20–23], SiO_2_- [24], graphite- [25, 26], and germanium- [27] based thin electrode films/substrate systems and obtained the lithiation-induced stresses in these electrodes. Different from the measurement of stress, the measurement of chemical strain during (de)lithiation is generally conducted on freestanding thin film electrodes using DIC. Composite electrodes based on graphite [28], lithium manganese oxide (LMO) [29], V_2_O_5_ [30], and MWCNTs/V_2_O_5_ [31] have been studied using DIC to analyze the distribution and evolution of chemical strain. The lithiation-induced strain is highly sensitive to the Li concentration, and in general, the higher the Li concentration is, the greater the chemical strain will be. Tavassol et al. combined in situ chemical stress and chemical strain measurements of graphite composite electrodes during electrochemical cycling and introduced the electrochemical stiffness of an electrode. Their results show that electrochemical stiffness changed dramatically due to the formation of different lithium-graphite intercalation compounds during cycling [32].

Although great progress has been achieved in the investigation of Li-induced chemical strain and stress, temperature-dependent in situ chemical strain has not been experimentally investigated yet. Temperature plays an extremely important role in the electrochemical performance of LIBs. At high temperatures, the increase in the redox rate and the enhancement in the reaction of lithium salt with solvent in electrolyte lead to the deterioration of the cycling stability of LIBs [33, 34]. In this work, a homemade device is used to in situ measure the chemical strain and nominal capacity in a freestanding graphite-based composite electrode during lithiation/delithiation cycling. The chemical in-plane strains of the graphite electrode during cycling at different temperatures and charging/discharging rates were characterized with the DIC method. As expected, the in situ measurements indicate that lithiation induces tensile strain in the electrode and the strain level is governed by the lithium content inserted. The chemical strain during high-temperature cycling is lower than that during low-temperature cycling at the same nominal state of charge (SOC), while the chemical strain is nearly independent of the charging/discharging rate in the range of 0.1C to 0.4C. With in situ measured charging/discharging curves, especially the residual strain and residual capacity, and the EIS spectra, the analysis of chemical strains suggests that the electrolyte decomposition during cycling at various temperatures is the reason for the temperature-dependent variation in chemical strain. The experimental results reveal that the electrolyte decomposition is more severe at high temperatures. Furthermore, a novel electrochemical-mechanical model is developed to determine the strain caused by SEI and evaluate the partial molar volume of Li in SEI. It is found that the strain induced by SEI is ~18% of the total strain and ~35% of the residual strain at 20°C, which increases significantly to ~40% and ~62%, respectively, at 60°C. The strain caused by SEI is higher at a higher temperature, and the partial molar volume of Li in SEI decreases with increasing temperature, thereby implying the structural and compositional changes of SEI induced by temperature.

## 2. Results and Discussion

### 2.1. *In Situ* Measurement of the Chemical Strain

**Figure 1:**
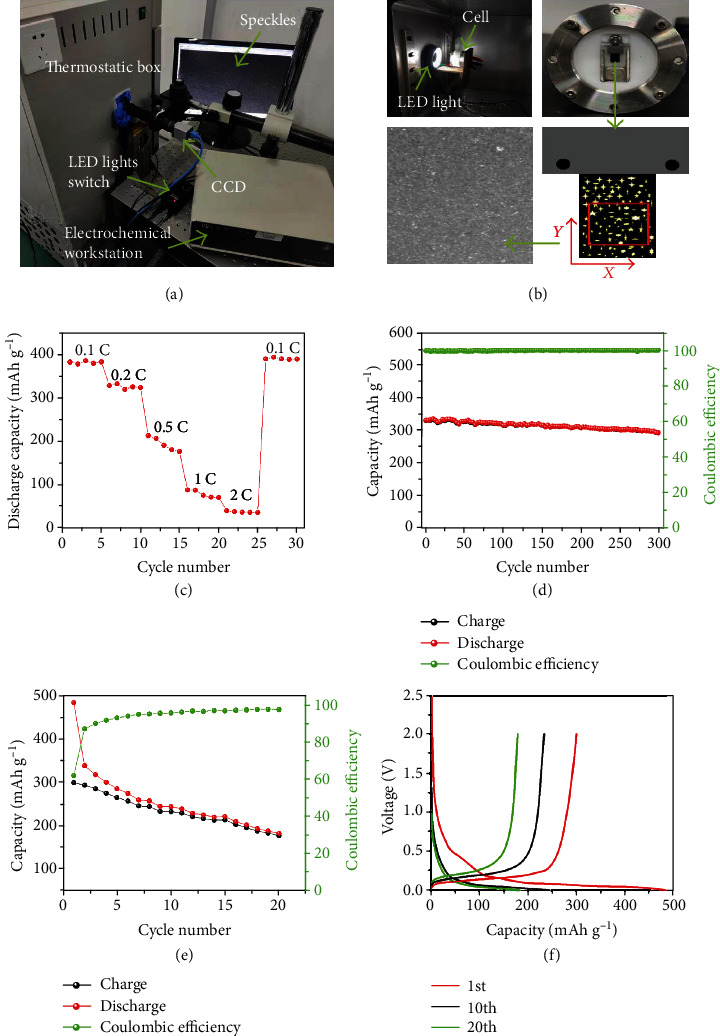
The homemade device for the chemical strain measurements. (a) Optical images of the chemical strain measurement device, including the CCD camera, electrochemical workstation, computer, and thermostatic box. (b) The homemade battery cell, a sketch of the cantilever electrode with one end fixed and electronically connected, region of interest (ROI), and the surface of the graphite composite electrode with natural speckles. (c) Rate performance and (d) cycling performance at charging/discharging rate 0.2C of the freestanding graphite-based composite electrode tested using conventional coin half-cells at room temperature. (e) Cycling performance at charging/discharging rate 0.2C and (f) charge-discharge curves of the selected cycles using the homemade device.

Figures 1(a) and 1(b) show the experimental configuration of the battery tester, homemade battery cell, and the camera and zoom lens for the DIC measurement, which is able to measure the two-dimensional displacement field and then strain field by matching the pictures before and after deformation. The up-end of the cantilever graphite electrode is spot welded on a stainless steel substrate, and the rest part of the electrode is immersed in electrolyte and freely expands/contracts during lithiation/delithiation. Optical images of the electrode surface with a size of 3 × 1.5 mm (outlined by the red rectangle in Figure 1(b)) are captured near the lower end of the electrode using the CCD camera. Figure 1(b) and Fig. S1 are representative images of the electrode surface, clearly showing the natural speckle pattern of the flat graphite electrode surface. Fig. S2 shows the initial charge-discharge potential profiles of the electrode in the coin half-cells at 0.1C. The initial discharge capacity of the electrode reaches ~385.9 mAh g^−1^ with a coulombic efficiency of ~81.3%. Figures 1(c) and 1(d) present the rate capacities and cycling performance of the graphite LIB anode in the coin half-cell at room temperature. The specific capacity of the electrode in the coin half-cell reaches ~371.5 mAh g^−1^ at 0.1C, which is comparable to the theoretical value of graphite [35, 36]. The specific capacity decreases obviously from 371.5 mAh g^−1^ to 324.2, 190.8, 76.2, and 39.1 mAh g^−1^ when increasing the rate from 0.1C to 0.2, 0.5, 1, and 2C, respectively. When the rate is reduced from 2C to 0.1C, the specific capacity recovers to ~370 mAh g^−1^, as shown in Figure 1(c). The electrode in the coin half-cell exhibits excellent cycling stability at 0.2C (Figure 1(d)). Fig. S3 shows that the areal capacity of the freestanding graphite anode is ~0.35 mAh cm^−2^. After 300 cycles at 0.2C, a reversible capacity of ~300 mAh g^−1^ was retained. Figures 1(e) and 1(f) present the cycling performance and charge-discharge curves of the cantilever graphite electrode in the homemade cell at room temperature. The charge-discharge curves of the cantilever graphite electrode are similar to those in the coin half-cell. Although the cycling performance of the cantilever graphite electrode is poor, its capacities in the initial 5 cycles are comparable to that in the coin cell. Fig. S4 shows the optical images of the graphite electrode at different lithiation states. The high capacity and stability at low rates in the initial several cycles and the flat surface with appropriate natural speckle patterns of the electrode ensure the feasibility and reliability of the chemical strain measurement by DIC.

**Figure 2:**
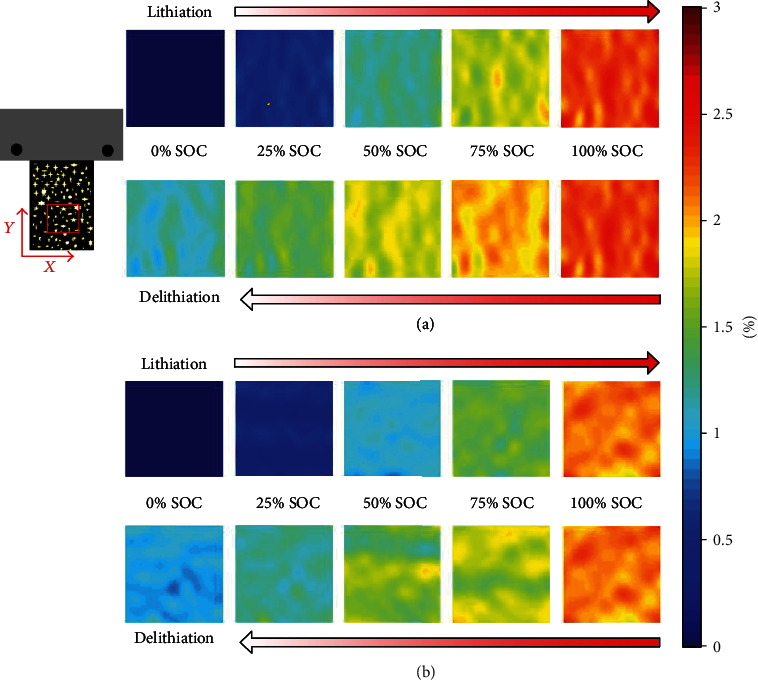
Strain contours of the freestanding graphite-based composite electrode. (a) Strain contours of *ε*_*xx*_ and (b) strain contours of *ε*_*yy*_ at different nominal states of charge (SOC) in the first lithiation/delithiation cycle at 0.2C with a size of 1.5 × 1.5 mm of the ROI.

The real pixel size is 2.5 *μ*m × 2.5 *μ*m, and the size of 20 × 20 pixels forms a domain, over which the average values of displacements and strains are taken to represent displacement and strain at the domain center. Fig. S5 shows the contour plots of the horizontal and vertical displacement, and Figure 2 shows the contour plots of the horizontal normal strain (*ε*_*xx*_) and vertical normal strain (*ε*_*yy*_) of the cantilever graphite electrode as a function of nominal SOC during cycling at 0.2C. The experimental results show that both *ε*_*xx*_ and *ε*_*yy*_ are approximately independent of the coordinate, i.e., spatially uniform. This is because the ROI is sufficiently far from the fixed end of the cantilever. For the same reason, the shear strain is negligible (Fig. S6). The strain contours in Figure 2 indicate that the two normal strains, *ε*_*xx*_ and *ε*_*yy*_, are always tensile when the virgin electrode before lithiation is taken as the undeformed state. In the lithiation process, both *ε*_*xx*_ and *ε*_*yy*_ magnitudes increase monotonically with nominal SOC to reach the peaks. Once delithiation happens, the tensile strain gradually decreases and residual tensile strain exists at the delithiation end, implying there are some irreversible electrochemical processes. Figure 2 illustrates that the magnitude of *ε*_*yy*_ is slightly lower than that of *ε*_*xx*_, which is due to cantilever electrode bending. To avoid any potential influence of cantilever electrode bending, the horizontal normal strain *ε*_*xx*_ is used to represent the isotropic deformation induced by lithiation/delithiation. Furthermore, horizontal normal strain *ε*_*xx*_ is averaged over the entire ROI, and the average ${\bar{\varepsilon }}_{xx}$ is used in the following analysis and discussion.

### 2.2. Strain Evolution during (De)Lithiation at Different Temperatures

**Figure 3:**
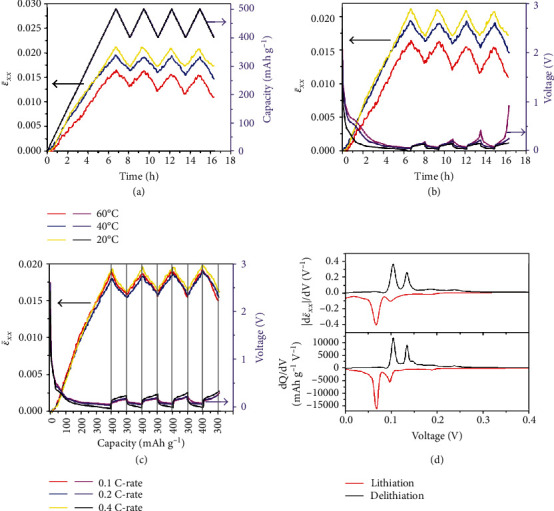
Strain evolution during cycling at different temperature/C-rate under limited capacity. (a) ${\bar{\varepsilon }}_{xx}$ and capacity versus time during cycling at different temperatures at a rate of 0.2C. The initial lithiation reaches 500 mAh g^−1^ and then delithiation to 400 mAh g^−1^ and then cycling between 500 and 400 mAh g^−1^. (b) ${\bar{\varepsilon }}_{xx}$ and voltage versus time during cycling at different temperatures at a rate of 0.2C. (c) ${\bar{\varepsilon }}_{xx}$ and voltage versus capacity during cycling at different rates and 20°C, where the capacity axis is separated into lithiation/delithiation segments. (d) Derivative of capacity and derivative of strain (${\bar{\varepsilon }}_{xx}$) with respect to voltage for the third cycle data.

In the in situ measurements at a constant charging/discharging rate, the voltage and the chemical strain are recorded versus time, and the nominal capacity is a linear function of time in charging or discharging.  Figure 3(a) shows ${\bar{\varepsilon }}_{xx}$ and nominal capacity versus time during cycling at the rate of 0.2C and temperatures of 20°C, 40°C, and 60°C, indicating clearly that the chemical strain and the nominal capacity reach peaks and valleys simultaneously, where the nominal capacity peak and valley values are set to be 500 mAh g^−1^ and 400 mAh g^−1^, respectively, in the present work. Fig. S7 shows that the variation of voltage is antiphase with that of nominal capacity. The variation of chemical strain in phase with that of nominal capacity indicates that the chemical strain is determined by the Li content in the electrode, and the Li content includes those inserted into the graphite lattice and those in SEI. Jones studied the effect of SEI on the strain of graphite electrode and found that the maximum strain measured in an electrolyte reduction-dominated test was ~0.2% [37]. Under the constant rate condition, Figures 3(a) and 3(b) illustrate that the chemical strain increases monotonically with decreasing temperature. Figs. S8 and S9 prove that the thermal expansion of a fresh graphite electrode and a lithiated graphite electrode is negligible when temperature increases from 20°C to 60°C. Thus, the smaller strain at higher temperatures at the same nominal SOC should be the consequence of more significant electrolyte decomposition, indicating that the lithium intercalated into graphite is factually less at high temperatures than low temperatures at the same nominal SOC [38, 39].  Figure 3(c) compares ${\bar{\varepsilon }}_{xx}$ of the graphite electrode cycled between 400 and 300 mAh g^−1^ at 0.1C, 0.2C, and 0.4C, at 20°C, indicating that there is no obvious difference among the strain curves at the three rates and the chemical strain of the graphite electrode is primarily correlated with the lithium concentration.  Figure 3(d) shows the derivative of capacity and the derivative of ${\bar{\varepsilon }}_{xx}$ with respect to the voltage with the data in the third cycle. The peaks in the capacity derivative are associated with specific phase transitions of graphite induced by Li intercalation and are in line with those reported by Dahn [40]. The peaks in the strain derivative almost replicate the location and relative magnitude of the peaks in the derivative of capacity, indicating that the reversible strain that developed at the macroscale in the graphite composite electrode is directly related to the atomic-scale changes in graphite layer spacing associated with different graphite-lithium intercalation compounds.

**Figure 4:**
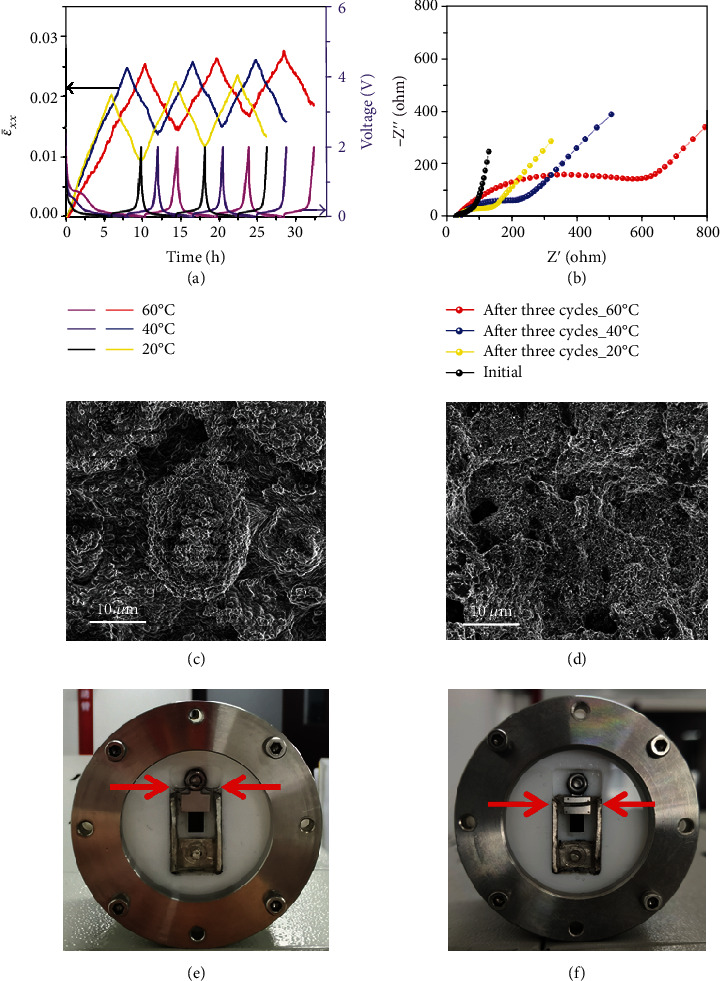
Strain evolution and electrolyte decomposition during cycling between 2 V and 0.01 V at different temperatures. (a) ${\bar{\varepsilon }}_{xx}$ and voltage versus time at a rate of 0.2C. (b) The electrochemical impedance spectroscopy (EIS) spectra of the homemade cells tested at 20°C before lithiation and after three cycles at different temperatures of 20°C, 40°C, and 60°C, respectively. (c, d) The SEM images of the cantilever electrode surface, (c) before lithiation and (d) after three lithiation/delithiation cycles at temperature 20°C. (e, f) The height of the electrolyte level in the homemade battery cell after three lithiation/delithiation cycles at temperatures of (e) 20°C and (f) 60°C.

Figure 4(a) shows the evolution of ${\bar{\varepsilon }}_{xx}$ and potential when the cell was fully charged and discharged at 0.2C for three cycles in the voltage range of 0.01-2 V vs. Li/Li^+^, at different temperatures. ${\bar{\varepsilon }}_{xx}$ reaches its maximum at the end of discharge and valley at the end of charge. Both the maximum ${\bar{\varepsilon }}_{xx}$ and discharge capacity are higher at a higher temperature, which is probably caused by more lithium intercalation into graphite and SEI formation. Increasing temperature will accelerate the electrolyte decomposition, resulting in more by-products and probably a larger charge transfer resistance. Figure 4(b) shows the EIS spectra at 20°C of the homemade cells which are after three lithiation/delithiation cycles at different temperatures. The cell cycled at 60°C has a much larger charge transfer resistance, which may originate from more significant electrolyte decomposition. In comparison to the cantilever electrode surface before lithiation (Figure 4(c)), the electrode surface after three lithiation/delithiation cycles at temperature 20°C manifests the formation of the solid electrolyte interface (SEI) layer, as shown in Figure 4(d). The formation of the SEI layer is more obvious at 40°C (Fig. S10b) and 60°C (Fig. S10c). Figures 4(e) and 4(f) show the heights of the electrolyte level after three cycles of lithiation/delithiation at temperatures 20°C and 60°C, respectively. The chamber of the in situ cell is completely filled with electrolytes before the discharging/charging cycle (Fig. S11). However, after three lithiation/delithiation cycles at temperature 60°C (Figure 4(f)), the height of the electrolyte level is lowered more significantly than that at temperature 20°C, indicating serious decomposition of the electrolyte during high-temperature cycling. The serious electrolyte decomposition is also reflected in the initial charge-discharge curves. Fig. S12 shows the nominal discharge capacity and the nominal charge capacity of each cycle at temperatures of 20, 40, and 60°C. It is found that the charge/discharge nominal capacity increases with raising temperature, and the charge capacity is significantly lower than the discharge capacity due to the electrolyte decomposition during discharge.

### 2.3. Chemical Strain Induced by SEI and Partial Molar Volume of Li

The measured chemical strain of the graphite composite electrode during discharging is attributed to two aspects: the lithium intercalation into graphite lattice (partially reversible) and the lithium consumed in the inactive materials which can be treated as the formation and growth of SEI (irreversible). In general, the measured linear strain *ε*(*n*_Li_) in the graphite composite electrode during lithiation is expressed as

(1) $$\begin{array}{c} \varepsilon \left(n_{\text{Li}}\right)=\frac{\int_{0}^{n_{\text{Li}}}{\bar{V}}_{\text{Li}}^{\text{li}}dn_{\text{Li}}}{3V_{0}}, \end{array}$$

where ${\bar{V}}_{\text{Li}}^{\text{li}}$ is the nominal partial molar volume of Li in the composite electrode during lithiation and *n*_Li_ is the total mole number of Li, which links linearly with the nominal capacity and is called the nominal mole number. The chemical strain of electrode during delithiation is given by

(2a) $$\begin{array}{c} \Delta \varepsilon_{\text{de}}=\left(\varepsilon_{\max}\left(n_{\text{Li}}^{\max}\right)-\varepsilon_{\text{de}}\right), \end{array}$$

where *ε*_max_(*n*_Li_^max^) is the chemical strain induced by the max Li mole number and

(2b) $$\begin{array}{c} \varepsilon_{\text{de}}=\frac{\int_{n_{\text{Li}}}^{n_{\text{Li}}^{\max}}{\bar{V}}_{\text{Li}}^{\text{deli}}dn_{\text{Li}}}{3V_{0}}, \end{array}$$

with ${\bar{V}}_{\text{Li}}^{\text{deli}}$ being the nominal partial molar volume of Li in the electrode during delithiation.

**Figure 5:**
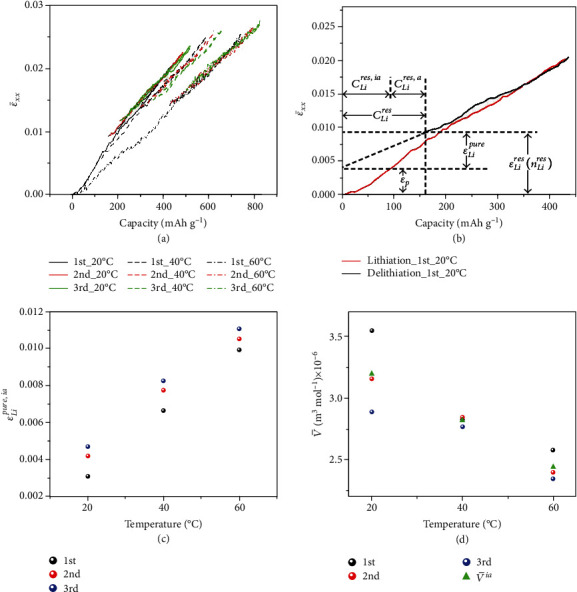
Determination of the strain induced by SEI and Li partial molar volume. (a) The chemical strain versus nominal capacity during cycling in the voltage range of 0.01-2 V vs. Li/Li^+^ at a rate of 0.2C and temperatures of 20, 40, and 60°C. (b) Determination of *ε*_Li_^res^ (*n*_Li_^res^), *ε*_*p*_(*n*_Li_ = 0), and *ε*_Li_^pure^ from the curves of strain versus nominal capacity. (c) The strain caused by SEI at temperatures of 20, 40, and 60°C. (d) The partial molar volume of Li in electrically inactive matrix (SEI), ${\bar{V}}_{\text{Li}}^{\text{ia}}$, of each cycle and the average partial molar volume of Li in electrically inactive matrix (SEI), ${\bar{V}}^{\text{ia}}$, at temperatures of 20, 40, and 60°C.

Figure 5(a) shows the curves of chemical strain versus nominal capacity during lithiation/delithiation cycling. After the first lithiation/delithiation cycle, the chemical strain does not completely recover to its initial value (that is 0 for the 1^st^ cycle), which leads to residual chemical strain *ε*_Li_^res^(*n*_Li_^res^) conjugated with residual Li mole number *n*_Li_^res^. The curve of strain versus nominal capacity (Li mole number) is approximately linear near the end of delithiation (Figure 5(a)), which allows one to extend the delithiation line of strain versus nominal capacity (Li mole number) to *n*_Li_ = 0. In this way, the residual chemical strain *ε*_Li_^res^(*n*_Li_^res^) is separated into two parts. The intercept gives the plastic strain *ε*_*p*_(*n*_Li_ = 0) generated in the lithiation/delithiation cycle, as illustrated in Figure 5(b) and Figs. S13 and S14. The other part is named the pure residual strain *ε*_Li_^pure^ caused by the residual Li in the composite electrode, i.e.,

(3a) $$\begin{array}{c} \varepsilon_{\text{Li}}^{\text{pure}}=\varepsilon_{\text{Li}}^{\text{res}}\left(n_{\text{Li}}^{\text{res}}\right)-\varepsilon_{p}\left(n_{\text{Li}}=0\right). \end{array}$$

The residual Li, *n*_Li_^res^, comprises the residual Li in the graphite lattice and the residual Li in the SEI. Thus, *ε*_Li_^pure^ can be expressed as the sum of the strain induced by SEI (*ε*_Li_^pure,ia^) and residual Li in graphite lattice (*ε*_Li_^pure,a^),

(3b) $$\begin{array}{c} \varepsilon_{\text{Li}}^{\text{pure}}=\varepsilon_{\text{Li}}^{\text{pure},\text{a}}+\varepsilon_{\text{Li}}^{\text{pure},\text{ia}}=\frac{{\bar{V}}_{\text{Li}}^{\text{a}}n_{\text{Li}}^{\text{res},\text{a}}+{\bar{V}}_{\text{Li}}^{\text{ia}}n_{\text{Li}}^{\text{res},\text{ia}}}{3V_{0}}, \end{array}$$

where ${\bar{V}}_{\text{Li}}^{\text{a}}$ and ${\bar{V}}_{\text{Li}}^{\text{ia}}$ are the partial molar volumes of Li in electrically active particles (graphite) and electrically inactive materials (SEI), respectively, and *n*_Li_^res,a^ and *n*_Li_^res,ia^ are the residual Li mole number in the active particles and inactive materials, respectively. The value of Li partial molar volume ${\bar{V}}_{\text{Li}}^{\text{a}}=4.17\times 10^{-6}$ m^3^ mol^−1^ in graphite is available in the literature [41]. At the preset voltage of 0.01 V, the maximum capacity of lithium insertion into graphite is 350 mAh g^−1^ [42], corresponding to a maximum Li mole number *n*_Li,max_^a^ = 2.09 × 10^−5^ mol in the studied electrode with the mass of the graphite being 1.6 mg. The residual mole number of Li staying in the graphite particles can be estimated from

(4) $$\begin{array}{c} n_{\text{Li}}^{\text{res},\text{a}}=n_{\text{Li},\max}^{\text{a}}-n_{\text{De}}^{i}, \end{array}$$

where *n*_De_^*i*^ is the mole number of Li extracted from graphite during delithiation and *i* (*i* = 1, 2, 3) is the cycle number. With the estimated value of *n*_Li_^res,a^ and the experimentally measured *n*_Li_^res^, the residual mole number of Li in the inactive component (SEI) is calculated by *n*_Li_^res,ia^ = *n*_Li_^res^ − *n*_Li_^res,a^. Then, the partial mole volume of Li in the SEI can be determined using Equation (3b). Table S1 summarizes the values of *ε*_Li_^pure^, *ε*_Li_^pure,a^, *ε*_Li_^pure,ia^, *ε*_*p*_(*n*_Li_ = 0), *ε*_Li_^res^(*n*_Li_^res^), and*n*_Li_^res^ after each cycle at different temperatures. As shown in Figure 5(c), the strain induced by SEI, *ε*_Li_^pure,ia^, increases with raising temperature, which is due to the more significant electrolyte decomposition and a larger volume of SEI formed at high temperature. Meanwhile, the contribution of SEI to the total strain is more at a higher temperature. It is found that the strain induced by SEI is ~18% of the total strain (~2.21%) after full lithiation and ~35% of the residual strain (~1.15%) after one cycle at 20°C, which increases significantly to ~40% of the total strain (~2.65%) and ~62% of the residual strain (~1.64%), respectively, at 60°C. The partial molar volumes of Li in SEI at different cycles and temperatures are calculated, and the average partial molar volume over the 3 cycles, ${\bar{V}}^{\text{ia}}$, is used to represent the practical partial molar volume of Li in SEI at a specific temperature, which is plotted in Figure 5(d), indicating the decrease in the partial molar volume of Li in SEI with increasing temperature. This might be attributed to the fact that the SEI possesses a porous structure at high temperature [43, 44]. The porous structure expands more as temperature increases and thus provides larger room to accommodate Li ions. As a result, the partial molar volume of Li in SEI decreases with increasing temperature.

## 3. Conclusions

In conclusion, a homemade in situ device is designed to measure chemical strain and nominal capacity simultaneously during electrochemical lithiation/delithiation of graphite electrodes at various temperatures and rates. The in situ measurements evaluate residual chemical strain and residual Li in both graphite lattice and SEI. It was found that the chemical strain of the electrode scaled proportionally to the lithium concentration in graphite. The increasing rate from 0.1C to 0.4C had no obvious influence on the strain of the electrode at the same nominal SOC. Raising temperature decreased the chemical strain of the electrode during lithiation at the same nominal SOC, as there were less lithium-ions inserted into graphite and more electrolyte decomposition at high temperatures. Furthermore, a new electrochemical-mechanical model is proposed to distinguish the strain induced by SEI and evaluate the partial molar volume of Li in the SEI layer at different temperatures. The SEI contributes significantly to the total strain during high-temperature cycling, and the “negative partial molar expansibility” implies the chemical and structural changes in the SEI layer induced by raising temperature. The strain measurements, combined with the electrochemical-mechanical model, reveal the larger residual strain and more significant SEI rearrangements at high temperatures which may degrade the electrochemical performance of graphite electrodes. Our experimental protocols and analytical method will enable more detailed investigations of electrode mechanics during electrochemical lithiation and electrolyte performance.

## 4. Materials and Methods

### 4.1. Materials

Graphite particles, carbon black, and carboxymethyl cellulose binder (CMC) were homogeneously mixed in a weight ratio of 8 : 1 : 1 in deionized water. The obtained slurry was coated onto copper foil using a doctor blade, followed by drying under ambient conditions. Then, the composite electrode was carefully peeled off of the copper foil, creating a freestanding electrode of 60-80 *μ*m thick, with no current collector. The freestanding electrode was cut using a razor blade into pieces approximately 6 mm × 4 mm and stored in an oven at 80°C prior to the DIC measurements.

### 4.2. Electrochemical Measurements

Coin cells (CR2032) were assembled using the freestanding electrode as the working electrode, Li metal as the counter electrode, and 1 M LiClO_4_ dissolved in ethylene carbonate and diethyl carbonate (1 : 1 by volume) as the electrolyte, in an argon-filled glove box, where the contents of water and oxygen were less than 0.1 ppm. The electrochemical cycling tests of the coin half-cells were performed using a battery testing system (Neware, BTS 4000) at 25°C, in the potential range of 0.01–2 V at a constant current of 0.2C (1C = 372 mA g^−1^). Cyclic voltammetry (CV) and electrochemical impedance spectroscopy (EIS) were measured using an electrochemical workstation (CHI 760E, China).

### 4.3. *In Situ* Strain Measurements

We designed a custom battery cell to enable in situ, full-field displacement/strain measurements during (de)lithiation of LIB electrodes. The cell contained a quartz window that allowed optical access to the electrode surface during cycling. Lithium metal was used as the counter electrode. The freestanding electrode was cantilevered from the edge of a stainless steel substrate and spot welded to the substrate at one end, creating a nearly unconstrained electrode. The custom cell was assembled in an argon-filled glove box, where the contents of water and oxygen were less than 0.1 ppm. The whole experimental equipment is schematized in Figure 1. The in situ cell was discharged and charged at a constant current at 20°C, 40°C, and 60°C, respectively. Noncontact DIC technique was used to measure in situ the displacement/strain fields of the graphite electrode surface. Composite graphite electrodes have a natural speckle pattern appropriate for DIC at large magnifications. A CCD camera with a resolution of 1624 × 614 pixels was used to in situ gather the dynamic movement images of speckle patterns on the graphite composite electrode surface. The sampling rate of the camera was set as 250 s per image in this work. A grid (step size of 20 pixels) was defined on the region of interest (ROI). The subset size was 20 × 20 pixels, and the scale factor was 2.5 *μ*m/pixels. The Matlab-based DIC code downloaded from open-source code by Jones [37] was used in this work to obtain the strain data. The details of the transformation for CCD images to the strain mapping results and the evaluation of the accuracy of the DIC code can be found in Reference [37]. A reference image was captured before cycling, and all displacement and strain calculations were computed with respect to the reference image.

### 4.4. Electrode Characterizations

The freestanding electrodes before and after cycling were characterized by SEM (Hitachi SU-8230). Before the electrode was mounted on the sample holder, the cell after 3 cycles was disassembled and the electrode was washed 3 times with anhydrous dimethyl carbonate and dried in the glove box to remove the residual electrolyte.

## Data Availability

The data used to support the findings of this study are available from the corresponding author upon request.

## Supplementary Materials

Supplementary Materials Fig. S1: characterization of electrode surface. Fig. S2: the initial charge-discharge potential profiles of the freestanding electrode at 0.1C. Fig. S3: the areal capacity of the graphite anode at 0.2C. Fig. S4: the optical images of the cantilever graphite electrode at different lithiation states. Fig. S5: displacement contours of the freestanding graphite-based composite electrode. Fig. S6: ${\bar{\varepsilon }}_{xx}$ and ${\bar{\varepsilon }}_{yy}$, shear strain ${\bar{\varepsilon }}_{xy}$, and the corresponding potential profiles during cycling at a rate of 0.2C. Fig. S7: the voltage and capacity versus time during cycling at different temperatures at a rate of 0.2C. Fig. S8: the strain evolution of a fresh graphite electrode during temperature increase from 5°C to 60°C. Fig. S9: the strain evolution of a lithiated graphite electrode when temperature increased from 20°C to 60°C. Fig. S10: the SEM images of the cantilever electrode surface after three lithiation/delithiation cycles. Fig. S11: the height of the electrolyte level in the homemade battery cell before and after three lithiation/delithiation cycles. Fig. S12: charge/discharge capacity of each cycle at different temperatures. Fig. S13: the residual strain. *ε*_Li_^res^(*n*_Li_^res^), *ε*_*p*_, and *ε*_Li_^pure^ after (a) the first cycle, (b) the second cycle, and (c) the third cycle at 20°C and 0.2C. Fig. S14: the residual strain. *ε*_Li_^res^(*n*_Li_^res^), *ε*_*p*_, and *ε*_Li_^pure^  after each cycle, (a–c) at 40°C and 0.2C and (d–f) at 60°C and 0.2C. Table S1: calculated material parameters of the graphite composite electrode. ${\bar{V}}^{\text{ia}}$ is the average value of ${\bar{V}}_{\text{Li}}^{\text{ia}}$ over the 3 cycles. Supplementary text: electrochemical-mechanical model.

## References

[1] Zheng J., Engelhard M. H., Mei D. (2017). Electrolyte additive enabled fast charging and stable cycling lithium metal batteries. *Nature Energy*.

[2] Birkl C. R., Roberts M. R., McTurk E., Bruce P. G., Howey D. A. (2017). Degradation diagnostics for lithium ion cells. *Journal of Power Sources*.

[3] Deng B., Shen L., Liu Y. (2017). Porous Si/C composite as anode materials for high-performance rechargeable lithium-ion battery. *Chinese Chemical Letters*.

[4] Zhang H., Li C., Piszcz M. (2017). Single lithium-ion conducting solid polymer electrolytes: advances and perspectives. *Chemical Society Reviews*.

[5] Qi Y., Guo H. B., Hector L. G., Timmons A. (2010). Threefold increase in the Young's modulus of graphite negative electrode during lithium intercalation. *Journal of the Electrochemical Society*.

[6] Li D., Wang Y., Hu J. (2018). Role of polymeric binders on mechanical behavior and cracking resistance of silicon composite electrodes during electrochemical cycling. *Journal of Power Sources*.

[7] Ankit V., Toshikazu K., Yuichiro T. (2018). Mechano-electrochemical interaction and degradation in graphite electrode with surface film. *Journal of the Electrochemical Society*.

[8] Chen J., Thapa A. K., Berfield T. A. (2014). _In-situ_ characterization of strain in lithium battery working electrodes. *Journal of Power Sources*.

[9] Sethuraman V. A., Chon M. J., Shimshak M., van Winkle N., Guduru P. R. (2010). _In situ_ measurement of biaxial modulus of Si anode for Li-ion batteries. *Electrochemistry Communications*.

[10] Xie H., Song H., Guo J. G., Kang Y., Yang W., Zhang Q. (2019). In situ measurement of rate-dependent strain/stress evolution and mechanism exploration in graphene electrodes during electrochemical process. *Carbon*.

[11] Haimei X., Haibin S., Yilan K. (2018). In situ experimental measurement of the mechanical properties of carbon-based electrodes during the electrochemical process. *Journal of the Electrochemical Society*.

[12] Jones E., Tavassol H., Gewirth A., White S. R., Sottos N. R. Modulus variation of composite graphite electrodes in lithium-ion batteries during electrochemical cycling.

[13] Wang Y., Zhang Q., Li D. (2018). Mechanical property evolution of silicon composite electrodes studied by environmental nanoindentation. *Advanced Energy Materials*.

[14] Kim S. J., Chang D., Zhang K., Graham G., van der Ven A., Pan X. (2018). Accordion strain accommodation mechanism within the epitaxially constrained electrode. *ACS Energy Letters*.

[15] Chen T., Zhao P., Guo X., Zhang S. (2017). Two-fold anisotropy governs morphological evolution and stress generation in sodiated black phosphorus for sodium ion batteries. *Nano Letters*.

[16] Mukhopadhyay A., Sheldon B. W. (2014). Deformation and stress in electrode materials for Li-ion batteries. *Progress in Materials Science*.

[17] Sethuraman V. A., Nguyen A., Chon M. J. (2013). Stress evolution in composite silicon electrodes during lithiation/delithiation. *Journal of the Electrochemical Society*.

[18] Li D., Wang Y. (2020). _In-situ_ measurements of mechanical property and stress evolution of commercial graphite electrode. *Materials and Design*.

[19] Li D., Wang Y., Hu J., Lu B., Cheng Y. T., Zhang J. (2017). In situ measurement of mechanical property and stress evolution in a composite silicon electrode. *Journal of Power Sources*.

[20] Kumar R., Woo J. H., Xiao X., Sheldon B. W. (2017). Internal microstructural changes and stress evolution in silicon nanoparticle based composite electrodes. *Journal of the Electrochemical Society*.

[21] Sethuraman V. A., Chon M. J., Shimshak M., Srinivasan V., Guduru P. R. (2010). In situ measurements of stress evolution in silicon thin films during electrochemical lithiation and delithiation. *Journal of Power Sources*.

[22] Sethuraman V. A., Srinivasan V., Bower A. F., Guduru P. R. (2010). In situ measurements of stress-potential coupling in lithiated silicon. *Journal of the Electrochemical Society*.

[23] Chen J., Yang L., Han Y. (2019). An in situ system for simultaneous stress measurement and optical observation of silicon thin film electrodes. *Journal of Power Sources*.

[24] Rakshit S., Tripuraneni R., Nadimpalli S. P. V. (2018). Real-time stress measurement in SiO_2_ thin films during electrochemical lithiation/delithiation cycling. *Experimental Mechanics*.

[25] Mukhopadhyay A., Tokranov A., Sena K., Xiao X., Sheldon B. W. (2011). Thin film graphite electrodes with low stress generation during Li- intercalation. *Carbon*.

[26] Mukhopadhyay A., Tokranov A., Xiao X., Sheldon B. W. (2012). Stress development due to surface processes in graphite electrodes for Li-ion batteries: a first report. *Electrochimica Acta*.

[27] Nadimpalli Siva P. V., Tripuraneni R. Stress response of germanium electrodes during lithiation/delithiation cycling.

[28] Yang W., Xie H., Shi B., Song H., Qiu W., Zhang Q. (2019). In-situ experimental measurements of lithium concentration distribution and strain field of graphite electrodes during electrochemical process. *Journal of Power Sources*.

[29] Çapraz Ö. Ö., Rajput S., White S., Sottos N. R. (2018). Strain evolution in lithium manganese oxide electrodes. *Experimental Mechanics*.

[30] Mao W., Wang Z., Li C. (2018). In-situ characterizations of chemo-mechanical behavior of free-standing vanadium pentoxide cathode for lithium-ion batteries during discharge-charge cycling using digital image correlation. *Journal of Power Sources*.

[31] Wang Z., Huang H., Zeng L. (2019). In-operando deformation studies on the mechano-electrochemical mechanism in free-standing MWCNTs/V_2_O_5_ lithium ion battery electrode. *Electrochimica Acta*.

[32] Tavassol H., Jones E. M. C., Sottos N. R., Gewirth A. A. (2016). Electrochemical stiffness in lithium-ion batteries. *Nature Materials*.

[33] Markovsky B., Rodkin A., Cohen Y. S. (2003). The study of capacity fading processes of Li-ion batteries: major factors that play a role. *Journal of Power Sources*.

[34] Araki K., Sato N. (2003). Chemical transformation of the electrode surface of lithium-ion battery after storing at high temperature. *Journal of Power Sources*.

[35] Shi H. (1998). Coke vs. graphite as anodes for lithium-ion batteries. *Journal of Power Sources*.

[36] Tarascon J. M., Armand M. (2001). Issues and challenges facing rechargeable lithium batteries. *Nature*.

[37] Jones E. M. C. (2015). *Mechanics of Lithium-Ion Battery Electrodes, [Ph.D. thesis]*.

[38] Zhuang Q. C., Wei G. Z., Dong Q. F. (2009). Influence of temperature on the performance of a graphite electrode. *Acta Physico-Chimica Sinica*.

[39] Zhuang Q. C., Chen Z. F., Dong Q. F., Jiang Y. X., Zhou Z. Y., Sun S. G. (2005). Effects of methanol contaminant in electrolyte on performance of graphite electrodes for Li-ion batteries studied via electrochemical impedance spectroscopy. *Chemical Journal of Chinese Universities-Chinese*.

[40] Dahn J. R. (1991). Phase diagram of LixC6. *Physical Review B*.

[41] Xiao X., Wu W., Huang X. (2010). A multi-scale approach for the stress analysis of polymeric separators in a lithium-ion battery. *Journal of Power Sources*.

[42] Huang S., Ren J., Liu R., Yue M., Huang Y., Yuan G. (2017). Enhanced electrochemical properties of natural graphite anode using promising cross-linked ionomer binder in Li-ion batteries. *New Journal of Chemistry*.

[43] Andersson A. M., Edström K. (2001). Chemical composition and morphology of the elevated temperature SEI on graphite. *Journal of the Electrochemical Society*.

[44] Rodrigues M., Sayed F. N., Gullapalli H., Ajayan P. M. (2018). High-temperature solid electrolyte interphases (SEI) in graphite electrodes. *Journal of Power Sources*.

