# Supplementary material for: Chemical Strain of Graphite-Based Anode during Lithiation and Delithiation at Various Temperatures
Source: Research (Wash D C). 2021 Oct 26;2021:9842391. doi: 10.34133/2021/9842391 (PMC8566194; doi:10.34133/2021/9842391)
Supplement: Supplementary Materials — Fig. S1: characterization of electrode surface. Fig. S2: the initial charge-discharge potential profiles of the freestanding electrode at 0.1C. Fig. S3: the areal capacity of the graphite anode at 0.2C. Fig. S4: the optical images of the cantilever graphite electrode at different lithiation states. Fig. S5: displacement contours of the freestanding graphite-based composite electrode. Fig. S6: ε¯xx and ε¯yy, shear strain ε¯xy, and the corresponding potential profiles during cycling at a rate of 0.2C. Fig. S7: the voltage and capacity versus time during cycling at different temperatures at a rate of 0.2C. Fig. S8: the strain evolution of a fresh graphite electrode during temperature increase from 5°C to 60°C. Fig. S9: the strain evolution of a lithiated graphite electrode when temperature increased from 20°C to 60°C. Fig. S10: the SEM images of the cantilever electrode surface after three lithiation/delithiation cycles. Fig. S11: the height of the electrolyte level in the homemade battery cell before and after three lithiation/delithiation cycles. Fig. S12: charge/discharge capacity of each cycle at different temperatures. Fig. S13: the residual strain. εLires(nLires), εp, and εLipure after (a) the first cycle, (b) the second cycle, and (c) the third cycle at 20°C and 0.2C. Fig. S14: the residual strain. εLires(nLires), εp, and εLipure after each cycle, (a–c) at 40°C and 0.2C and (d–f) at 60°C and 0.2C. Table S1: calculated material parameters of the graphite composite electrode. V¯ia is the average value of V¯Liia over the 3 cycles. Supplementary text: electrochemical-mechanical model. [file 9842391.f1.docx]

Supplementary Materials

Chemical Strain of Graphite-Based Anode during Lithiation and Delithiation at Various Temperatures

Zeyu Xu, Xiuling Shi, Xiaoqiang Zhuang, Zihan Wang, Sheng Sun, Kaikai Li^*^, Tong-Yi Zhang^*^

**Fig. S1.** Characterization of electrode surface. (a) Speckle pattern and (b) grayscale speckle pattern, the scale factor of which was 2.5 μm/pixels, and (c-d) the SEM images of the electrode surface (c) before and (d) after cycling.


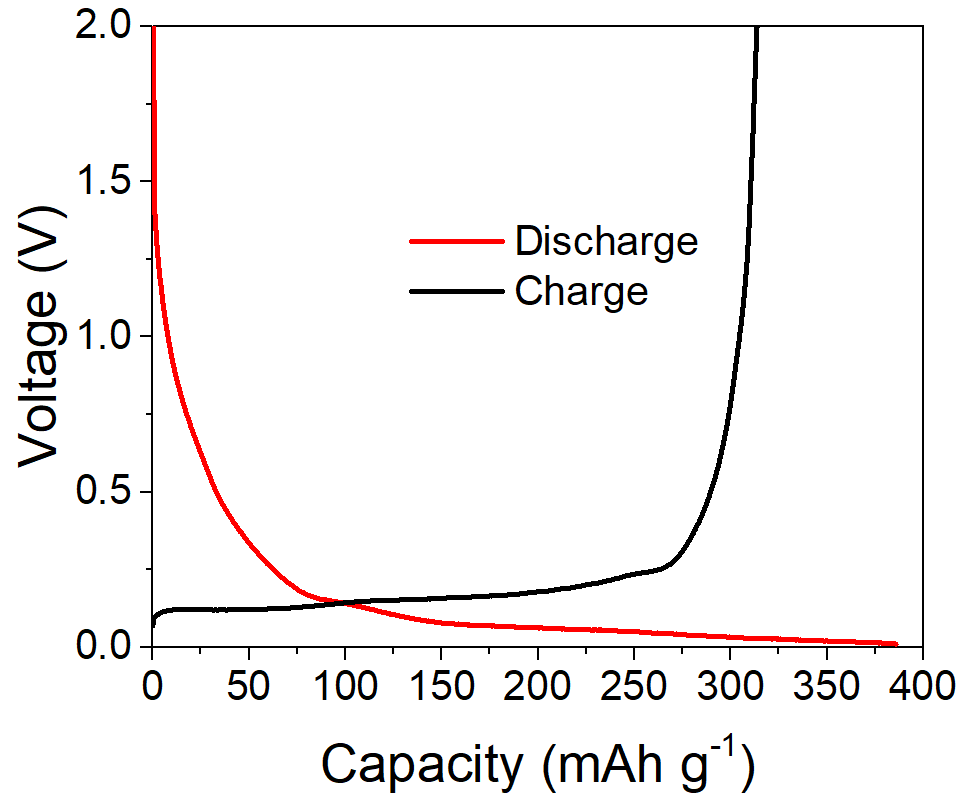


**Fig. S2.** The initial charge-discharge potential profiles of the freestanding electrode at 0.1 C.


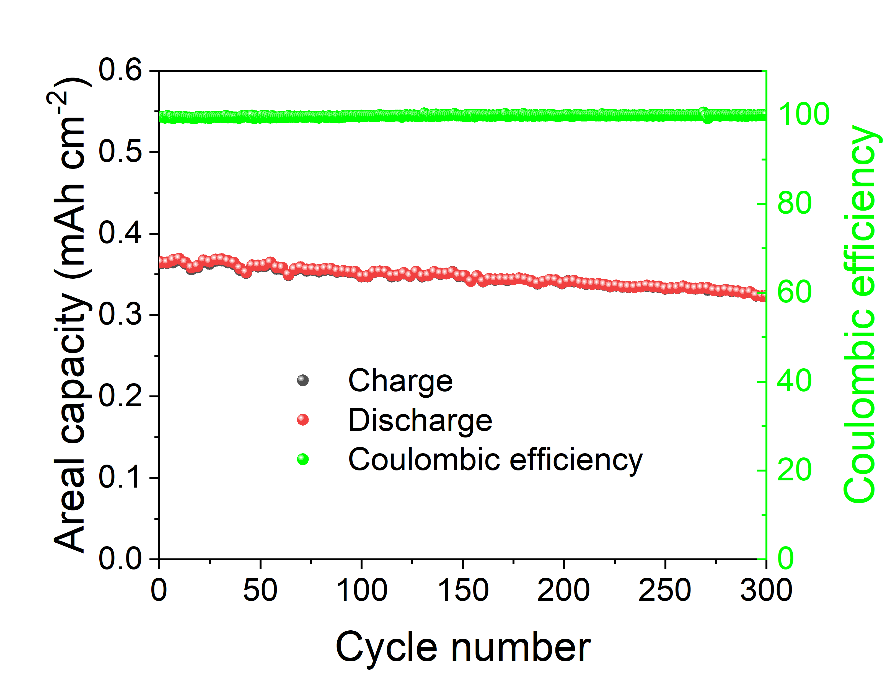


**Fig. S3.** The areal capacity of the graphite anode at 0.2 C.


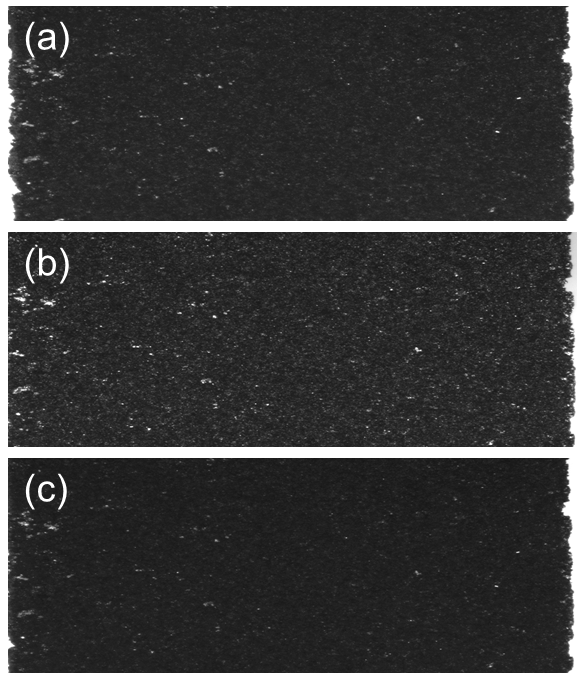


**Fig. S4.** The optical images of the cantilever graphite electrode at different lithiation state (a) pristine, (b) fully lithiated, and (c) fully delithiated.

**Fig. S5.** Displacement contours of the freestanding graphite-based composite electrode. Contour plots of (a) displacement along the *xx* direction and (b) displacement along the *yy* direction at different states of charge (SOC) in the first lithiation-delithiation cycle at 0.2 C with a size of 1.5 × 1.5 mm.


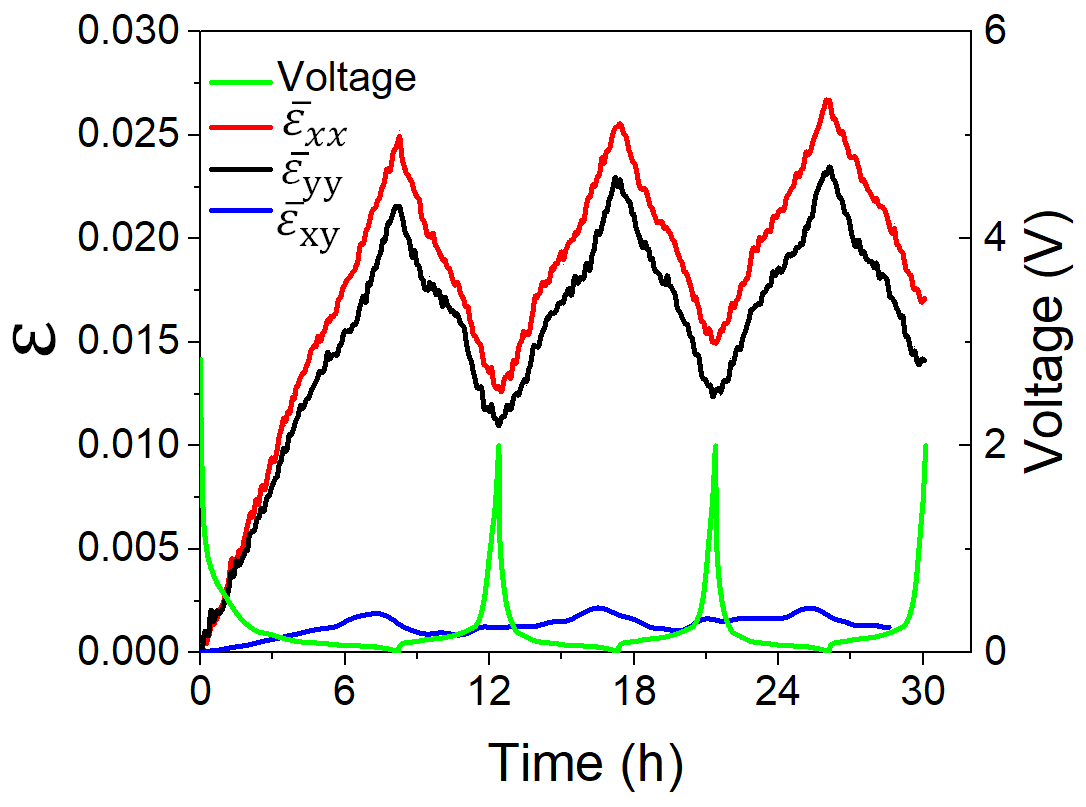


**Fig. S6.** The $\bar{\varepsilon}_{xx}$ and $\bar{\varepsilon}_{yy}$, shear strain $\bar{\varepsilon}_{xy}$, and the corresponding potential profiles during cycling at a rate of 0.2 C.

**
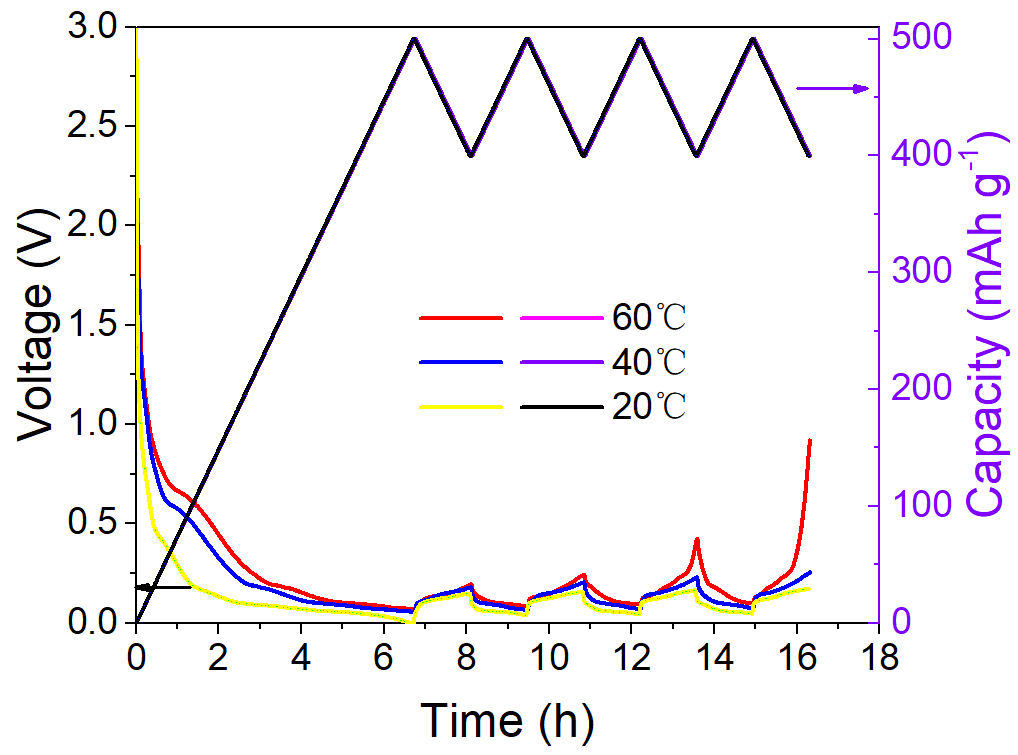
**

**Fig. S7.** The voltage and capacity versus time during cycling at different temperatures at a rate of 0.2 C.


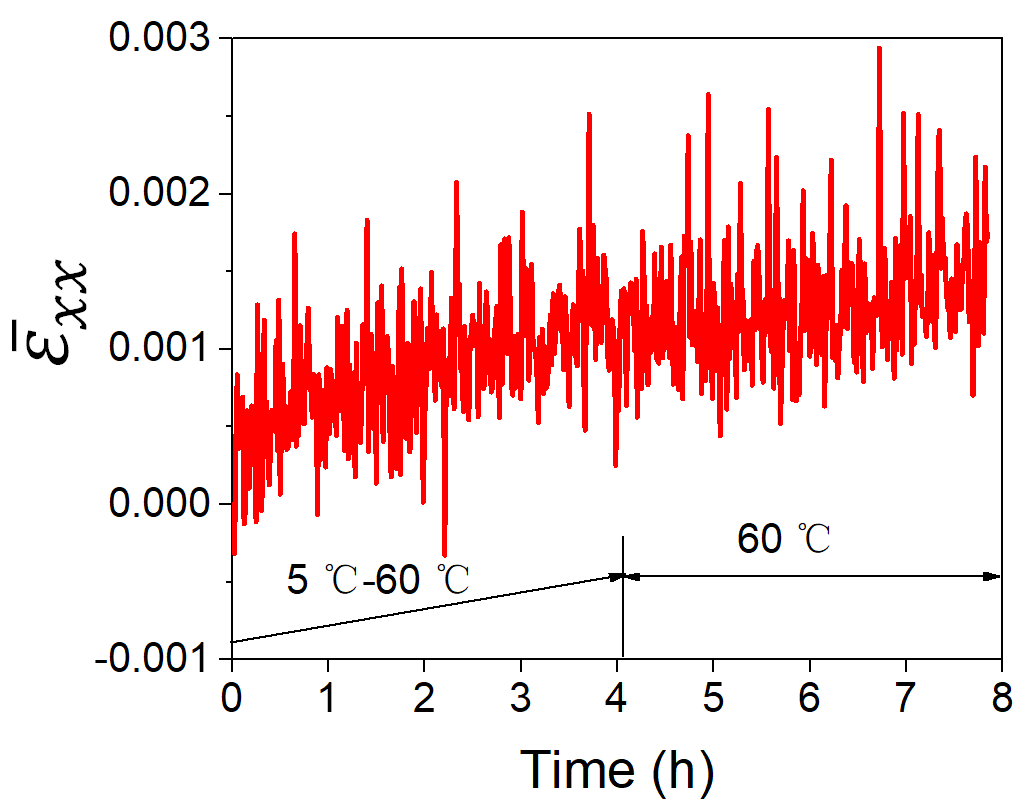


**Fig. S8.** The strain evolution of a fresh graphite electrode during temperature increase from 5 °C to 60 °C.


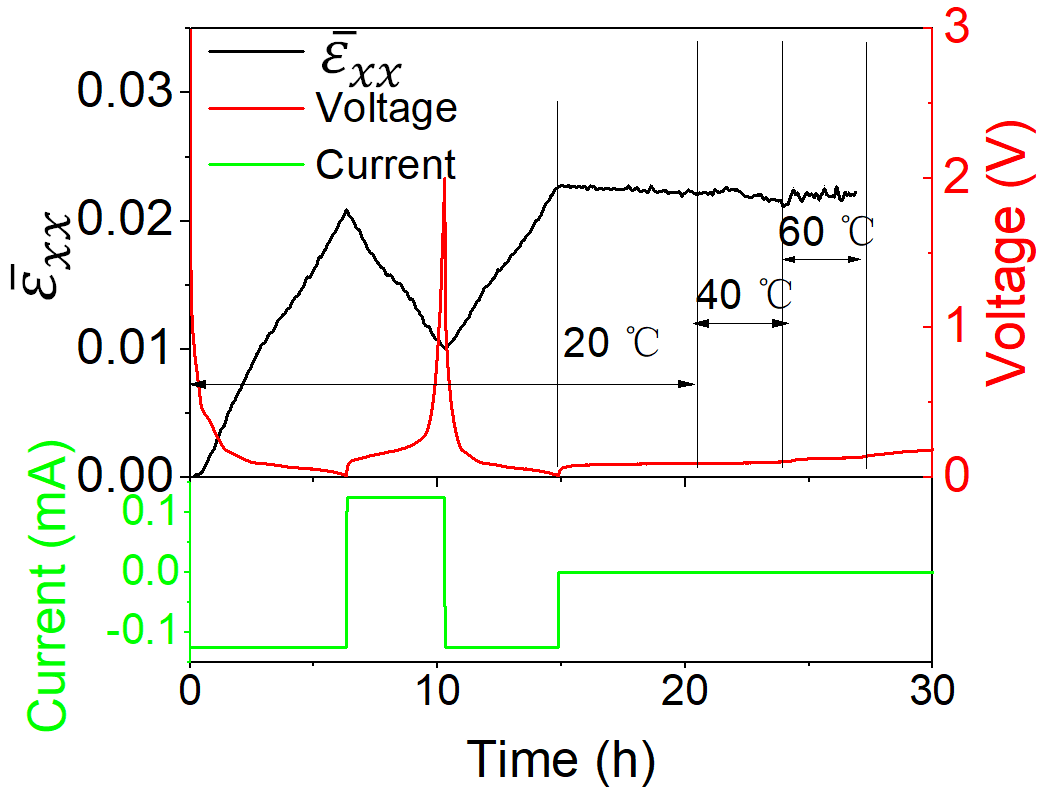


**Fig. S9.** The strain evolution of a lithiated graphite electrode when temperature increased from 20 °C to 60 °C.


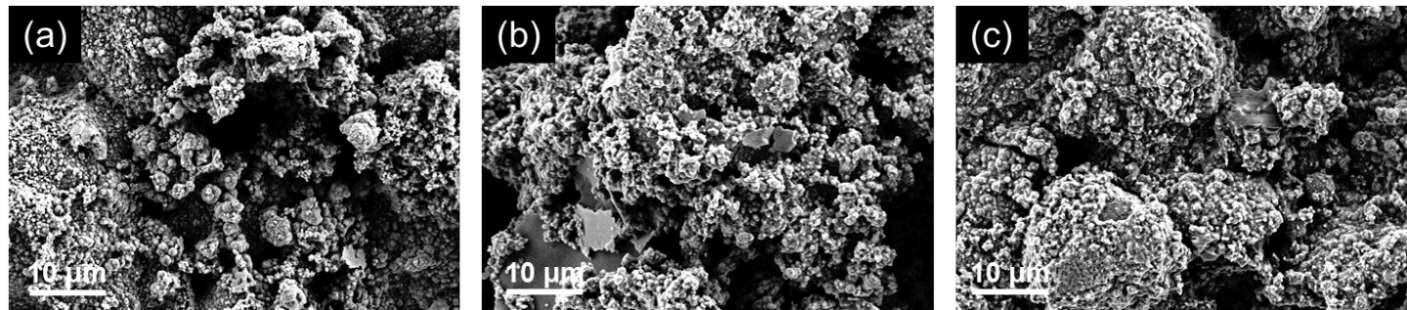


**Fig. S10.** The SEM images of the cantilever electrode surface after three lithiation/delithiation cycles at temperature (a) 20 °C, (b) 40 °C, and (c) 60 °C.


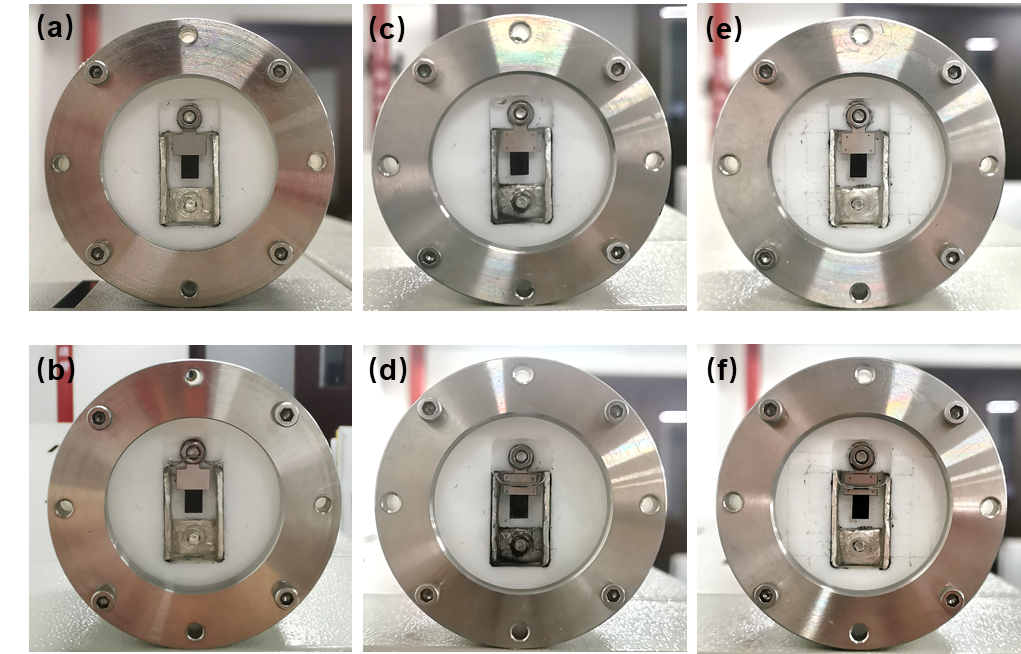


**Fig. S11.** The height of the electrolyte level in the home-made battery cell before (up) and after (down) three lithiation/delithiation cycles at temperature of (a, b) 20 °C, (c, d) 40 °C, and (e, f) 60 °C.

**Fig. S12.** Charge/discharge capacity of each cycle at different temperature. (a) The nominal discharge capacity and (b) the nominal charge capacity of each cycle at temperatures of 20, 40, and 60 °C.

**Fig. S13.** The residual strain. $\varepsilon_{Li}^{res}\left( n_{Li}^{res} \right)$, $\varepsilon_{p}$, and $\varepsilon_{Li}^{pure}$ after (a) the first cycle, (b) the second cycle, and (c) the third cycle at 20 ℃ and 0.2C.

**Fig. S14.** The residual strain. $\varepsilon_{Li}^{res}\left( n_{Li}^{res} \right)$, $\varepsilon_{p}$, and $\varepsilon_{Li}^{pure}$ after each cycle, (a-c) at 40 ℃ and 0.2C, and (d-f) at 60 ℃ and 0.2C.

**Table S1.** Calculated material parameters of the graphite composite electrode. $\bar{V}^{ia}$ is the average value of the $\bar{V}_{Li}^{ia}$ over the 3 cycles.

| Temperature | $\varepsilon_{Li}^{res}\left( n_{Li}^{res} \right)$ | $\varepsilon_{p}\left( n_{Li}=0 \right)$ | $\varepsilon_{Li}^{pure}$ | $C_{Li}^{res}$  (mAh/g) | $C_{Li}^{res,a}$  (mAh/g) | $C_{Li}^{res,ia}$  (mAh/g) | $\varepsilon_{Li}^{pure,a}$ | $\varepsilon_{Li}^{pure,ia}$ | $\bar{V}_{Li}^{ia}$  （×10^-6^） | $\bar{V}^{ia}$  （×10^-6^） |
| --- | --- | --- | --- | --- | --- | --- | --- | --- | --- | --- |
| 20 ℃_1th | 0.0093 | 0.0036 | 0.0056 | 158.5 | 64.3 | 94.2 | 0.00249 | 0.00311 | 3.548 | 3.198 |
| 20 ℃_2nd | 0.0117 | 0.0049 | 0.0068 | 210.2 | 66.9 | 143.3 | 0.00259 | 0.00421 | 3.157 |  |
| 20 ℃_3rd | 0.0134 | 0.058 | 0.0076 | 250.1 | 74.2 | 175.9 | 0.00288 | 0.00472 | 2.888 |  |
| 40 ℃_1th | 0.0138 | 0.0049 | 0.0088 | 308.5 | 55.2 | 253.3 | 0.00214 | 0.00666 | 2.828 | 2.813 |
| 40 ℃_2nd | 0.0150 | 0.005 | 0.0100 | 351.4 | 57.8 | 293.6 | 0.00224 | 0.00776 | 2.843 |  |
| 40 ℃_3rd | 0.0158 | 0.0052 | 0.0106 | 381.3 | 60.4 | 320.9 | 0.00234 | 0.00826 | 2.768 |  |
| 60 ℃_1th | 0.0142 | 0.0031 | 0.0111 | 444.9 | 30.4 | 414.5 | 0.00118 | 0.00992 | 2.575 | 2.436 |
| 60 ℃_2nd | 0.0166 | 0.0043 | 0.0123 | 518.5 | 46.2 | 472.3 | 0.00179 | 0.01051 | 2.393 |  |
| 60 ℃_3rd | 0.0185 | 0.005 | 0.0135 | 572 | 61.8 | 510.2 | 0.0024 | 0.0111 | 2.341 |  |

**Electrochemical-mechanical model:**

The total mole number of Li, $n_{Li}$, measured by the charging/discharging current and time, is called the nominal mole number. The chemical strain of electrode during lithiation is given by

$e\left( n_{Li} \right)=\frac{\int_{0}^{n_{Li}} \bar{V}_{Li}^{li}dn_{Li}}{V_{0}}$ (S1a)

where $\bar{V}_{Li}^{li}$ is the nominal partial molar volume of Li in the electrode during lithiation. The measured linear strain $\varepsilon$ is one third of the volumetric strain $\varepsilon=\frac{e}{3}$, i.e.,

$\varepsilon\left( n_{Li} \right)=\frac{\int_{0}^{n_{Li}} \bar{V}_{Li}^{li}dn_{Li}}{3V_{0}}$ (S1b)

The chemical strain includes reversible and irreversible strains. The chemical strain of electrode during delithiation is given by

$\Delta\varepsilon_{de}=\left( \varepsilon_{max}\left( n_{Li}^{max} \right)-\varepsilon_{de} \right)$ (S2a)

where $\varepsilon_{max}\left( n_{Li}^{max} \right)$ is the chemical strain induced by the max Li mole number and

$\varepsilon_{de}=\frac{\int_{n_{Li}}^{n_{Li}^{max}} \bar{V}_{Li}^{deli}dn_{Li}}{3V_{0}}$ (S2b)

with $\bar{V}_{Li}^{deli}$ being the nominal partial molar volume of Li in the electrode during delithiation. The delithiation ends at $n_{Li}^{min}$ and the value of $n_{Li}^{min}$ may depend on the cycle number. The experimentally measured value of $n_{Li}^{min}$ is actually the residual Li mole number $n_{Li}^{res}$ and the experimentally measured value of chemical strain conjugated with $n_{Li}^{res}$ is called the residual strain $\varepsilon_{Li}^{res}\left( n_{Li}^{res} \right)$. The curve of strain versus capacity (Li mole number) is approximately linear during delithiation (Fig. 5a). Extending the line of strain versus capacity (Li mole number) during delithiation to $n_{Li}=0$, the intercept gives the plastic strain $\varepsilon_{p}\left( n_{Li}=0 \right)$ generated in this charging/discharging cycle (Fig. 5b, Fig. S8 and Fig. S9). Thus, the residual Li mole number induces the purely residual strain $\varepsilon_{Li}^{pure}$

$\varepsilon_{Li}^{pure}=\varepsilon_{Li}^{res}\left( n_{Li}^{res} \right)-\varepsilon_{p}\left( n_{Li}=0 \right)$ (S3)

A part of the residual Li ions stays in the electrically active particles and the other part stays in the electrically inactive matrix (forming SEI), i.e.,

$n_{Li}^{res}=n_{Li}^{res,a}+n_{Li}^{res,ia}$ (S4a)

$C_{Li}^{res}=C_{Li}^{res,a}+C_{Li}^{res,ia}$ (S4b)

$n_{Li}^{res,a}=\frac{C_{Li}^{res,a}m_{a}}{F}$ (S4c)

$n_{Li}^{res,ia}=\frac{C_{Li}^{res,ia}m_{a}}{F}$ (S4d)

where superscripts “*a*” and “*ia*” denote electrically active and inactive, respectively. The *“F”* is the Faraday constant. The $m_{a}$=1.6 mg is the mass of the electrically active particles (graphite) in the studied electrode.

As measured in our coin cells, the maximum capacity of lithium insertion into graphite is assumed to be $C_{Max}^{a}$ = 350 mAh g^-1^, corresponding to a maximum Li mole number of $n_{Li, max}^{a}=\frac{C_{Max}^{a}m_{a}}{F}$= 2.09×10^-5^ mol in the studied electrode. Then, the $C_{Li}^{res,a}$ and $n_{Li}^{res,a}$can be estimated from

$C_{Li}^{res,a}=C_{Max}^{a}-C_{De}^{i}$ (S5a)

$n_{Li}^{res,a}=n_{Li,max}^{a}-n_{De}^{i}$ (S5b)

$\varepsilon_{Li}^{pure,a}=\frac{\bar{V}_{Li}^{a}n_{Li}^{res,a}}{3V_{0}}=\frac{\bar{V}_{Li}^{a}C_{Li}^{res,a}m_{a}}{3V_{0}F}$ (S6a)

$\varepsilon_{Li}^{pure,ia}=\frac{\bar{V}_{Li}^{ia}n_{Li}^{res,ia}}{3V_{0}}=\frac{\bar{V}_{Li}^{ia}C_{Li}^{res,ia}m_{a}}{3V_{0}F}$ (S6b)

where $\bar{V}_{Li}^{a}$ is the partial molar volume of Li in electrically active particles. Here, the electrically active particle is graphite and its $\bar{V}_{Li}^{a}$ can be found in the literature[1].

The partial molar volume of Li in electrically inactive matrix $\bar{V}_{Li}^{ia}$ can be estimated from

$\varepsilon_{Li}^{pure}=\frac{\bar{V}_{Li}^{a}n_{Li}^{res,a}+\bar{V}_{Li}^{ia}n_{Li}^{res,ia}}{3V_{0}}$ (S7)

References

1. Jones EMC (2015) Mechanics of lithium-ion battery electrodes. PhD. diss., University of Illinois, Urbana-Champaign.
